# The Newly Discovered Cytokine IL-34 Is Expressed in Gingival Fibroblasts, Shows Enhanced Expression by Pro-Inflammatory Cytokines, and Stimulates Osteoclast Differentiation

**DOI:** 10.1371/journal.pone.0081665

**Published:** 2013-12-10

**Authors:** Elisabeth A. Boström, Pernilla Lundberg

**Affiliations:** 1 Karolinska Institutet, Division of Periodontology, Department of Dental Medicine, Stockholm, Sweden; 2 Umeå University, Department of Molecular Periodontology, Umeå, Sweden; University of Toronto, Canada

## Abstract

**Background:**

Interleukin-34 (IL-34) is a recently discovered cytokine functionally overlapping macrophage colony stimulating factor (M-CSF), a mediator of inflammation and osteoclastogenesis in bone-degenerative diseases such as rheumatoid arthritis. The objective of this study was to assess the expression of IL-34 in human gingival fibroblasts and investigate if the pro-inflammatory cytokines tumor necrosis factor alpha (TNF-α) and Interleukin-1*Β* (IL-1β) modulate its expression, and moreover if IL-34 could contribute to recruitment of bone-resorbing osteoclasts.

**Methods:**

IL-34 expression was evaluated in gingival fibroblasts by real time PCR following stimulation by TNF-α, IL-1β, and treatment with inhibitors of intracellular pathways. The formation of osteoclasts was evaluated by tartrate-resistant acid phosphatase (TRAP) staining of bone marrow macrophages treated with IL-34 or M-CSF in addition to receptor activator of nuclear factor kappa-B ligand (RANKL).

**Results:**

IL-34 was expressed in gingival fibroblasts. The expression was enhanced by TNF-α and IL-1β, regulated by the transcription factor nuclear factor kappa B (NF-κ*Β*) and activation of c-Jun N-terminal kinase (JNK). Further, IL-34 supports RANKL-induced osteoclastogensis of bone marrow macrophages, independently of M-CSF.

**Summary:**

In conclusion, this study shows for the first time IL-34 expression in human gingival fibroblasts, stimulated by TNF-α and IL-1β, key mediators of periodontal inflammation. Furthermore, IL-34 can be substituted for M-CSF in RANKL-induced osteoclastogenesis. IL-34 may contribute to inflammation and osteoclastogenesis in bone-degenerative diseases such as periodontitis.

## Introduction

Periodontitis is an inflammatory disease of periodontal tissues leading to progressive bone resorption and ultimately tooth loss. In the inflamed periodontium, gingival fibroblasts, the most abundant cell type, drives inflammation by release of leukocyte-recruiting cytokines. The infiltrating leukocytes stimulate bone resorption through differentiation and activation of bone-resorbing osteoclasts via secretion of receptor activator of NF-κ*Β* ligand (RANKL), and colony-stimulating factors (CSF). CSFs are involved in macrophage-activation, inflammation, and osteoclastogenesis in periodontitis and other bone-degenerative diseases such as rheumatoid arthritis (RA). Thus, they are instrumental in mediating inflammation and tissue destruction.

Macrophage-CSF (M-CSF, CSF-1) is the primary regulator of survival, proliferation and differentiation of monocytes, macrophages, myeloid and osteoclast progenitor cells. M-CSF is consecutively expressed in gingival fibroblasts [1]. M-CSF binds to its receptor CSF1R (c-fms, CD115) on osteoclast progenitor cells, and in concert with RANKL binding to the RANK-receptor on the same cell, leads to differentiated and activated bone resorbing osteoclasts. Moreover, M-CSF drives macrophage-mediated inflammation. CSFs are known to modulate disease and inflammation and to play an important role in bone destruction [2]. M-CSF depletion is beneficial in blocking inflammation in animal models of periodontitis [3] and RA [4], [5] whereas M-CSF administration exacerbates inflammation and tissue destruction [4]. Moreover, CSF1 gene expression is associated with aggressive periodontitis [6]. Thus, further understanding of CSF-1R signaling and its modulatory effect on cells involved in periodontal inflammation will lead to deepened knowledge, and possibly new therapeutic strategies.

IL-34, an alternative ligand for CSF1R, was recently identified [7]. IL-34 shares important functions of M-CSF and regulates myeloid cell survival, differentiation, and proliferation [7]. IL-34 can substitute M-CSF in osteoclastogenesis [8] however, the current understanding of IL-34 in inflammatory bone-degenerative diseases is limited to a couple of reports concerning RA. IL-34 is elevated in serum and synovial fluid of RA patients [9], and is expressed in synovial tissue [10], [11]. Fibroblasts in RA are, similarly to in periodontitis, active cells highly important in the inflammatory process via modulation of myeloid cells leading to osteoclast activation and bone destruction. IL-34 expression was recently shown in synovial fibroblasts [10], [11], regulated by TNF-α and IL-1β, cytokines also known as important mediators of periodontal inflammation and bone destruction [12]. Moreover, IL-34 expression was shown in inflammatory oral tissue, related to the expression of TNF-α, IL-1β, IL-17, and IL-23 in Sjögren’s Syndrome [13], [14]. The role of IL-34 in periodontal disease, and its expression in gingival fibroblasts is yet unknown.

In this study, we hypothesized that IL-34 was expressed by gingival fibroblasts, the most abundant cell type in inflamed periodontium, and that the expression was regulated by TNF-α and IL-1β, known as key mediators of periodontal inflammation. We next explored the involvement of NF-κ*Β* and the mitogen-activated protein kinase (MAPK) signaling pathways in the regulation of IL-34 and M-CSF expression in gingival fibroblasts. Furthermore, we investigated if IL-34 could contribute to osteoclast recruitment.

## Materials and Methods

### Fibroblast Cultures

Gingival fibroblasts were isolated as previously described [15] from gingival papillar explants obtained from three clinically and systemically healthy voluntary donors, whose rights were protected by the local Ethical Committee of Umeå University, Umeå, Sweden, who approved the study. Written consents were received.

Gingival explants were placed at the bottom of culture dishes 60 cm^2^ (Nunc, Roskilde, Denmark) with α-MEM (α modification of Minimum Essential Medium) supplemented with 10% foetal calf serum (FCS, GIBCO-BRL/Life Technologies, Paisley, UK), L-glutamine (GIBCO-BRL/Life Technologies, Paisley, UK) and antibiotics (Meda AB, Solna, Sweden, and SIGMA-ALDRICH, St. Louis, USA ), referred to as basic medium, and left untouched for 7–10 days until outgrowth of fibroblasts from the explants was observed. The fibroblasts were then detached and seeded at a density of 3.5×10^4^ cells/cm^2^ and cultured until cells were 80–90% confluent. Media was changed and cells were incubated in the absence (controls) or presence of test substances for 48 h or for time-course experiment as indicated in the figure legends. Cells used in the experiments demonstrated a fibroblastic morphology and cells from passages 4–7 were used in the experiments.

### Cultures of Bone Marrow Macrophages (BMM)

CsA mice from the inbred colony at the animal facility unit at Umeå University were used. Animal care and experiments were approved and made in accordance with accepted standards of humane animal care and use, as considered appropriate by the Animal Care and Use Committee of Umeå University, Umeå, Sweden. The mice were kept on a 12-hour light/12-hour dark cycle and were fed standard chow and water ad libitum. Male mice at the age of 5–9 weeks were sacrificed by cervical dislocation and used for the experiments. Bone marrow cells were isolated from femurs of CsA mice as previously described [16]. Briefly, bone marrow cells were incubated for 2 h in tissue culture plastic dishes, after which the non-adherent cells were collected, centrifuged, resuspended in α-MEM with 10% FBS, L-glutamine, 100 U/ml bensylpenicillin, 100 µg/ml streptomycin, 100 µg/ml gentamycin sulphate, and seeded at 10^6^ cells/cm^2^ on cover slips in 24-well plates. After incubation with either M-CSF 25 ng/ml (R&D Systems, Abdingdon, UK) and RANKL 4 ng/ml (R&D Systems, Abdingdon, UK), or IL-34 (25, 50 and 100 ng/ml, R&D Systems, Abdingdon, UK) and RANKL (4 ng/ml) for 4 days, the cells were fixed and stained for tartrate resistant acid phosphatase (TRAP).

### TRAP Staining

TRAP staining was performed by use of the Leukocyte acid phosphatase kit (Sigma-Aldrich, Steinheim, Germany) according to manufacture’s instructions. TRAP^+^ cells with three or more nuclei were considered osteoclasts and the number of multinucleated osteoclasts was counted in a light microscope.

### RNA Isolation and First-stranded cDNA Synthesis

Total RNA from gingival fibroblast cell cultures was isolated by using the RNAqueous™–4PCR kit (Ambion, Austin, TX) according to instructions by the manufacturer. High Capacity cDNA Reverse Transcription Kit (Foster City, CA) was used to transcribe mRNA to cDNA.

### Quantitative Real-time Polymerase Chain Reaction (qPCR)

Taq-man (API PRISM 7900HT Sequence Detection System) was used to detect and analyze gene expression. The mRNA levels of *IL-34, M-CSF*, and *IL-6* were analyzed using specific primers/fluorescent probe mix from Applied Biosystems, Foster City, CA, USA. To rule out DNA contamination, samples in which the reverse transcription reaction had been omitted were also submitted to the PCR reaction, yielding no amplification. To control variability in amplification due to differences in starting mRNA-concentrations, *h-RPL-13a* was used as house-keeping gene.

### Statistical Analysis

The statistical analyses were performed using one-way analysis of variance (ANOVA) with Levene’s homogenicity test, and post-hoc Bonferroni’s or where appropriate Dunnett’s. Results are expressed as means ± standard error of means (SEM). SEM is shown when the height of the error bar is larger than the radius of the symbol.

## Results

### High Constitutional Expression of IL-34 in Gingival Fibroblasts

We evaluated the basal expression of IL-34 mRNA in human gingival fibroblasts. RT-PCR analysis showed that 3/3 individuals expressed IL-34 (Table 1). Moreover, the expression of IL-34 was 24–270 times the expression of IL-6, and 25–118 times the expression of M-CSF.

**Table 1:** IL-34 is constitutionally expressed at high levels in gingival fibroblasts.

	IL-34 (Qt)	IL-6 (Qt)	M-CSF (Qt)
GF 5	10.0	0.4	0.4
GF 6	9.4	0.4	0.08
GF 8	5.4	0.02	0.06

Basal mRNA expression of IL-34, M-CSF, and IL-6 in fibroblasts isolated from three individuals. Cells were cultured for 48 hrs and mRNA levels assessed by RT-PCR and normalized to those of hRPL-13a.

### Time Dependent Regulation of IL-34 Expression by TNF-α and IL-1β in Gingival Fibroblasts

**Figure 1:**
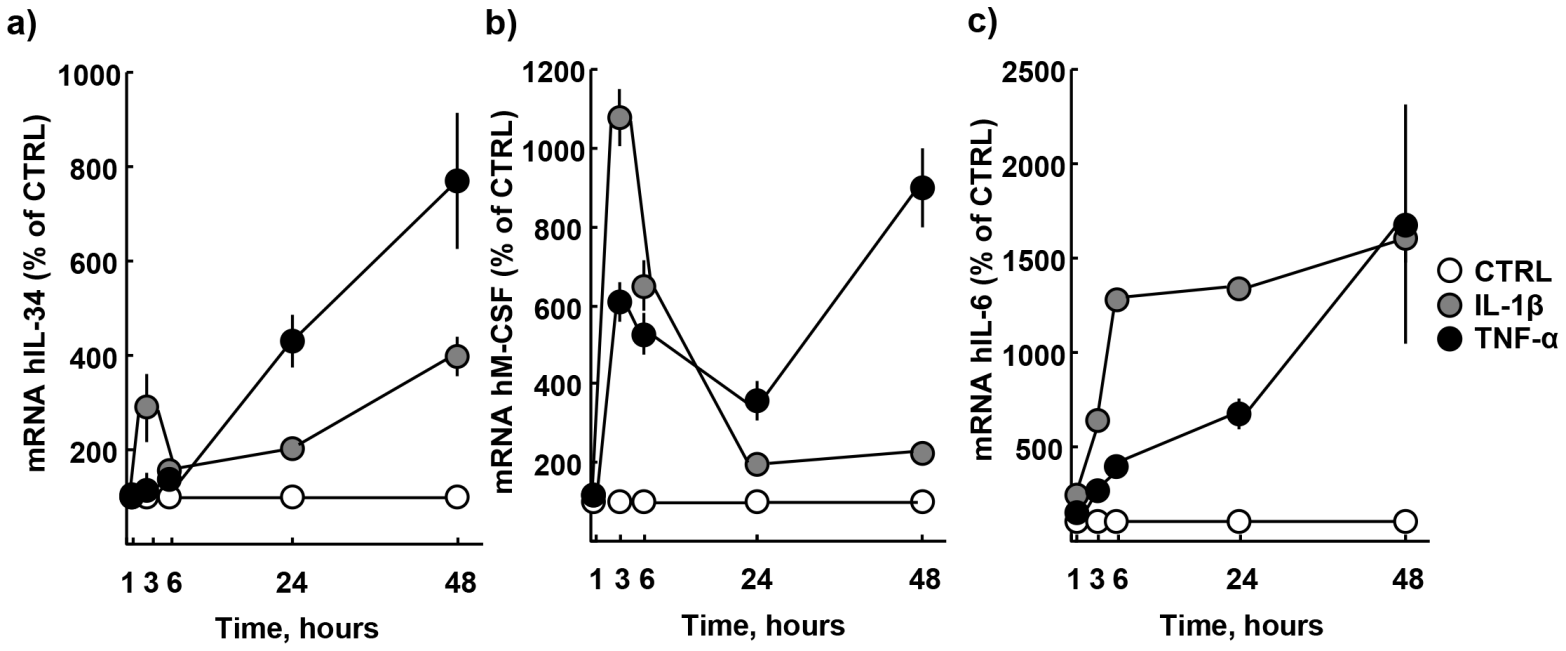
Time-dependent increase of IL-34, M-CSF, and IL-6 expression in gingival fibroblasts by TNF-α and IL-1β. (**A**) TNF-α (50 ng/ml) and IL-1β (100 pg/ml) stimulate IL-34, (**B**) M-CSF, and (**C**) IL-6 expression in a time-dependent manner. Data expressed as means ± SEM. Data represent three individual experiments.

We assessed the time dependent expression of IL-34 mRNA in gingival fibroblasts in response to the pro-inflammatory cytokines TNF-α and IL-1β. Cells were cultured at increasing time-points (1 h, 3 h, 6 h, 24 h, 48 h) in the absence or presence of TNF-α (50 ng/ml) or IL-1β (100 pg/ml). RT-PCR analysis showed a time-dependent up-regulation of IL-34 expression with an 8x maximum fold-change by TNF-α (bold dots, Fig. 1a), and a 4x maximum fold-change by IL-1β (grey dots, Fig. 1a) at 48 hrs. The expression of M-CSF was enhanced 9x by TNF-α (bold dots, Fig. 1b), and 2x by IL-1β at 48 hrs (grey dots, Fig. 1b). Parallel analysis of IL-6 expression showed a 17x maximum fold-change by TNF-α (bold dots, Fig. 1c), and a 16x maximum fold-change by IL-1β (grey dots, Fig. 1c) at 48 hrs.

### Concentration Dependent Regulation of IL-34 Expression by TNF-α and IL-1β in Gingival Fibroblasts

**Figure 2:**
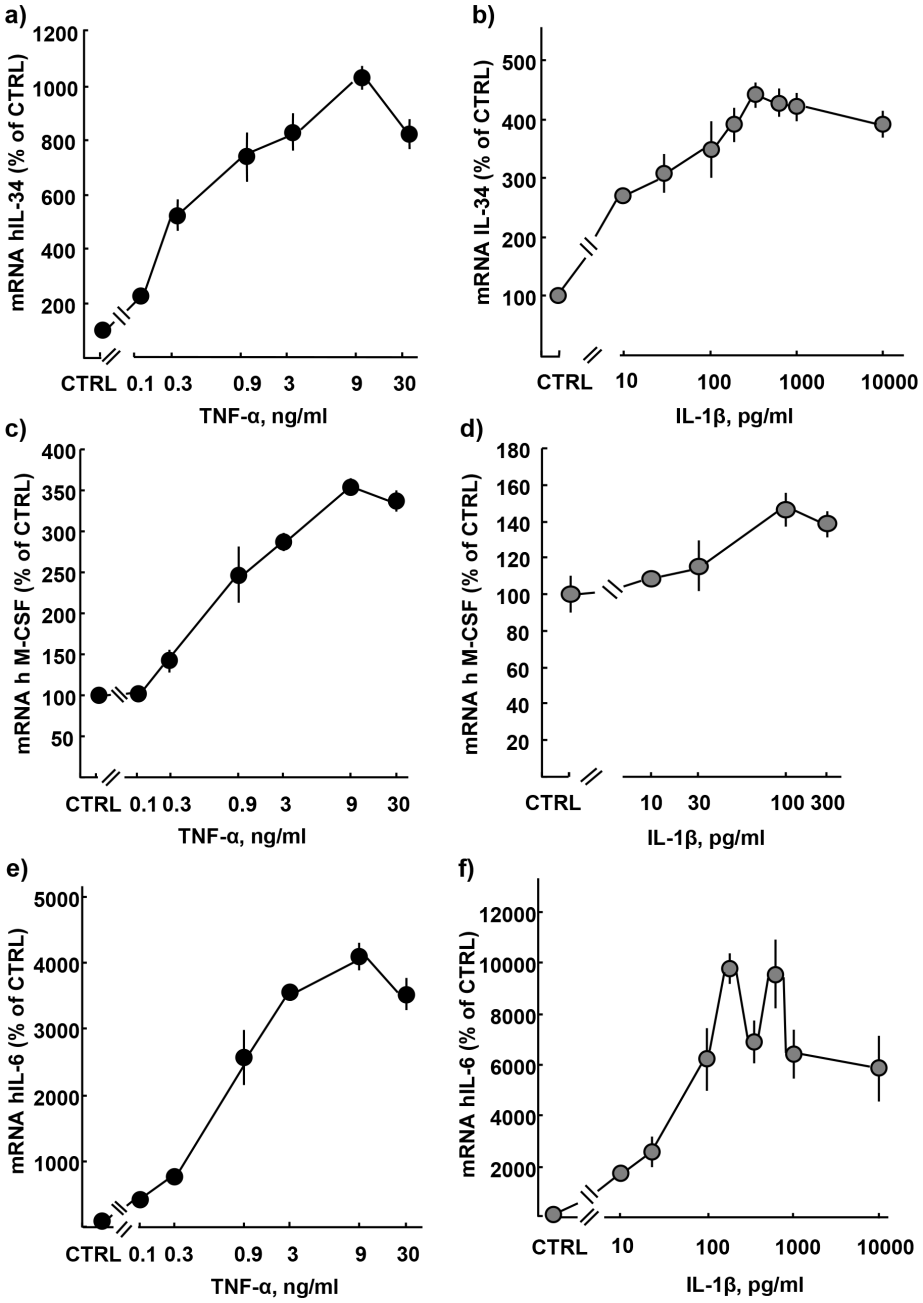
Dose-dependent increase of IL-34, M-CSF, and IL-6 expression in gingival fibroblasts by TNF-α and IL-1β. (**A**) TNF-α and (**B**) IL-1β increase IL-34 expression in a dose-dependent manner. (**C**) TNF-α and (**D**) IL-1β increase M-CSF expression in a dose-dependent manner. (**E**) TNF-α and (**F**) IL-1β increase IL-6 expression in a dose-dependent manner. Analysis performed at 48 h of incubation. Data expressed as means ± SEM. Data represent three individual experiments.

IL-34 expression peaked at 48 h following TNF-α and IL-1β treatment. Therefore, this time-point was used to assess the concentration dependent regulation of IL-34 expression by TNF-α (0.1–30 ng/ml) and IL-1β (10–10000 pg/ml). IL-34 expression was up-regulated in a concentration-dependent manner in response to TNF-α, with a 10x maximum fold-change at 9 ng/ml (EC50 = 0,3 ng/ml, Fig. 2a), and by IL-1β with a 4.5x maximum fold-change at 500 pg/ml (EC50 = 3 pg/ml, Fig. 2b). M-CSF expression was also concentration-dependently enhanced by TNF-α with a 3.5x maximum fold-change at 9 ng/ml (EC50 = 0.5 ng/ml, Fig. 2c), and by IL-1β with a 1.5x maximum fold-change at 100 pg/ml (EC50 = 50 pg/ml, Fig. 2d). Parallel analysis of IL-6 expression showed, as expected, a concentration-dependent up-regulation by TNF-α with a 41x maximum fold-change at 9 ng/ml (EC = 0,6 ng/ml, Fig. 2e), and by IL-1β with a 100x maximum fold-change at 500 pg/ml a (EC50 = 0,07 ng/ml, Fig. 2f).

### NF-κ*Β* and JNK are Required for TNF-α and IL-1β Stimulated IL-34 Expression in Gingival Fibroblasts

**Figure 3:**
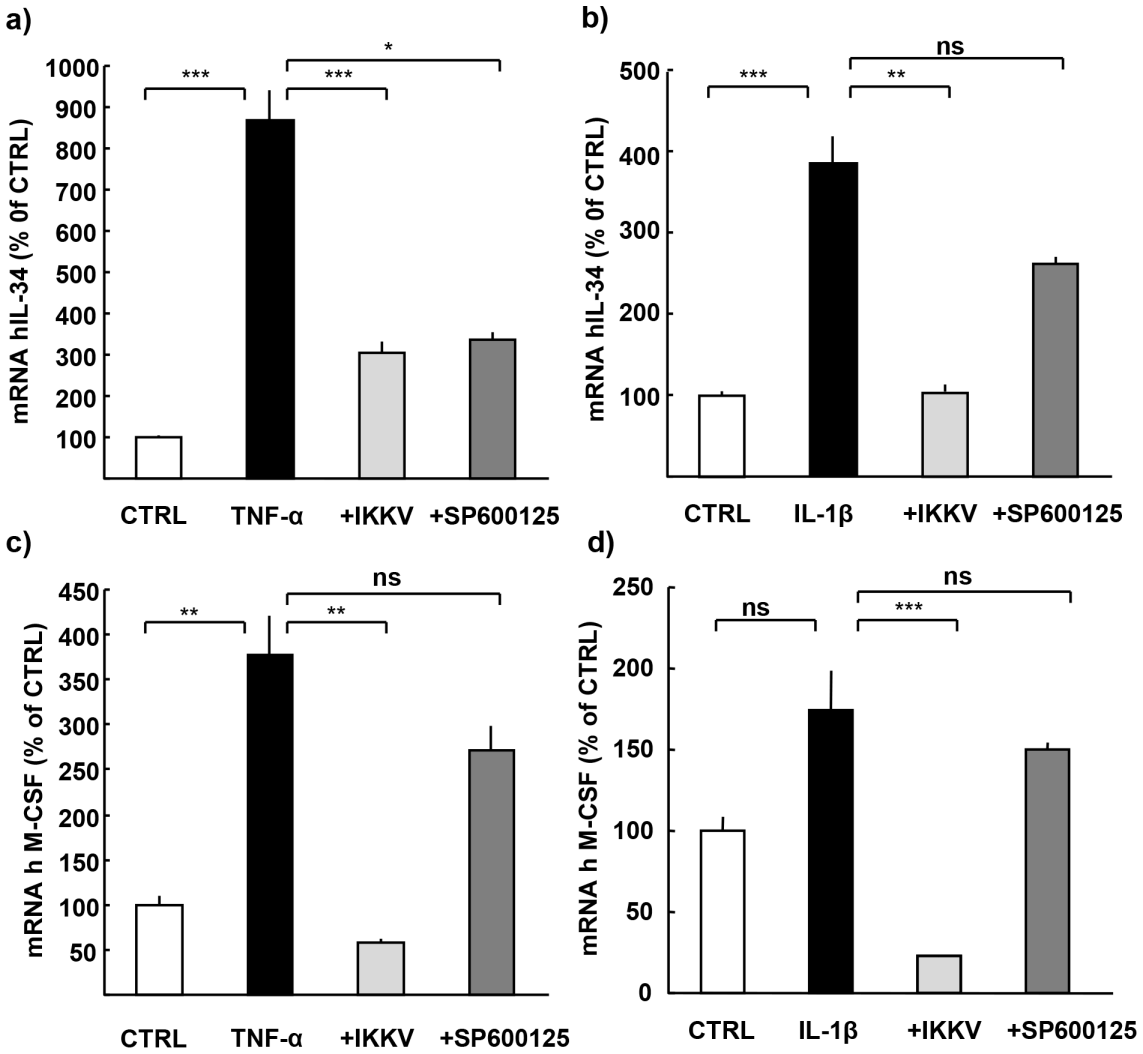
Involvement of NF-κΒ and JNK pathways in TNF-α and IL-1β stimulated IL-34 and M-CSF in gingival fibroblasts. (**A**) Down regulation of TNF-α stimulated IL-34 by IKKβ inhibitor V (IKKV) and JNK inhibitor and down regulation of (**B**) IL-1β stimulated IL-34 by IKKβ inhibitor V (IKKV). (**C**) Down regulation of TNF-α and (**B**) IL-1β stimulated M-CSF by IKKβ inhibitor V (IKKV). Analysis performed at 48 h of incubation. Data expressed as means ± SEM. Data represent three individual experiments.

NF-κ*Β* and the MAPK c-Jun N-terminal Kinase (JNK) are the two main signaling pathways activated by TNF-α and IL-1β in gingival fibroblasts [17]. To investigate the regulation of TNF-α and IL-1β stimulated IL-34 expression, involvement of these pathways was evaluated at 48 h. TNF-α (10 ng/ml) enhanced IL-34 expression (Fig. 3a). Addition of the specific IKKβ inhibitor (IKKV, 10 uM) or addition of the JNK inhibitor (SP600125, 10 uM) both significantly inhibited IL-34 expression (66% inhibition by IKKV versus 62% by SP600125). IL-1β significantly enhanced IL-34 expression (Fig. 3b). Addition of the IKKβ inhibitor significantly reduced IL-34 expression, notably almost to the level of non-treated cells (74% inhibition by IKKV). M-CSF expression was enhanced by TNF-α, and abolished by the IKKβ inhibitor (Fig. 3c). M-CSF expression was increased by IL-1β and abolished by the IKKβ inhibitor (Fig. 3d). The inhibitors alone had no effect on the mRNA levels of IL-34 or M-CSF (data not shown). Taken together, TNF-α stimulated IL-34 is regulated via NF-κ*Β* and JNK pathways, and IL-1β stimulated IL-34 is regulated via the NF-κ*Β* pathway. TNF-α and IL-1β stimulated M-CSF is regulated via NF-κ*Β*.

### IL-34 can Substitute M-CSF in RANKL-stimulated Osteoclast Formation

**Figure 4:**
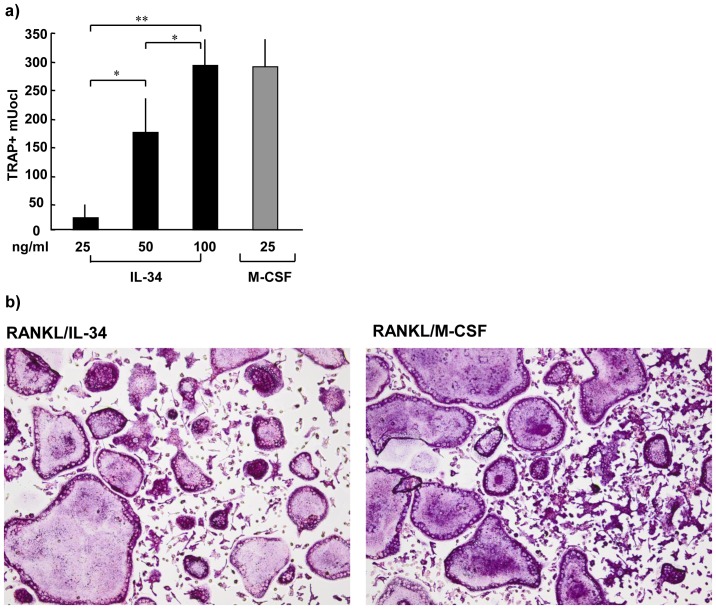
IL-34 substitutes M-CSF in RANKL induced osteoclastogenesis. (**A**) IL-34, together with RANKL, induces osteoclastogenesis of bone marrow macrophages in a concentration-dependent manner, to comparable levels of M-CSF/RANKL. (**B**) Representative photos (20X) of RANKL (4 ng/ml)/IL-34 (100 ng/ml) versus RANKL (4 ng/ml)/M-CSF (25 ng/ml) at day 4. Data expressed as means ± SEM.

To elucidate the role of IL-34 in osteoclastogenesis we studied the combined effect of IL-34 and RANKL on the formation of TRAP^+^ multinucleated osteoclasts *in vitro*. Multinucleated osteoclasts (288±76) were formed by day 4 of culture in mouse bone marrow macrophage cultures stimulated with 25 ng/ml M-CSF and 4 ng/ml RANKL (Fig. 4a and b). Addition of IL-34 at a concentration of 100 ng/ml together with RANKL (4 ng/ml) rendered in approximately the same number of osteoclasts as in response to M-CSF and RANKL (291±46, Fig. 4a and 4b). A concentration-dependent increase of osteoclast formation was observed in response to IL-34 comparing 25 ng/ml to 50 ng/ml, and 50 ng/ml to 100 ng/ml where addition of 50 ng/ml IL-34 together with RANKL (4 ng/ml) resulted in 40% less osteoclasts (172±56) compared to 100 ng/ml IL-34 (Fig. 4a). M-CSF or IL-34 *per se* gave no TRAP+ cells or osteoclasts (data not shown).

## Discussion

Pro-inflammatory cytokines promote inflammation, osteoclast formation and activity in bone-degenerative diseases such as rheumatoid arthritis and periodontitis. In the present study we demonstrate that the newly discovered cytokine IL-34 is expressed by gingival fibroblasts and that the pro-inflammatory cytokines TNF-α and IL-1β enhance its expression. To our knowledge this is the first report showing that resident cells in the gingiva express IL-34. It is intriguing because this molecule is associated with synovitis severity in RA, a periodontitis related disease [10]. Synovial fibroblasts do, as gingival fibroblast, express IL-34 and its expression is enhanced by TNF-α and IL-1β. The fact that IL-34 can entirely substitute M-CSF in RANKL-induced osteoclastogenesis that we and others have shown [8] is intriguing and pinpoints a potential pivotal role for IL-34 in inflammation-driven bone resorption.

The abundance of fibroblasts in gingival tissue [18], [19] together with the fact that synovial fibroblasts can secrete a variety of cytokines and enzymes involved in remodeling of bone and cartilage in rheumatoid arthritis [20] indicates the importance to investigate gingival fibroblast expression of molecules of importance in joint disease. We and others have earlier shown that gingival fibroblasts can secrete osteotropic IL-6 type cytokines, including IL-6, IL-11 and LIF [12]. Moreover, increased IL-6 expression is previously demonstrated in response to TNF-α and IL-1β [21], [22], where the stimulatory patterns of IL-6 shown in this study are in accordance with the data by Palmqvist et al [12]. Elevated IL-6 mRNA expression was demonstrated in fibroblasts from inflammatory gingival tissue compared to cells from healthy gingiva [23], [24] and studies have also demonstrated increased IL-6 levels in gingival crevicular fluid of pathological gingival pockets compared to controls [25]–[27]. Regarding IL-34, more studies are required to investigate if IL-34 is increased in gingival crevicular fluid in periodontally diseased sites, and if it is present in inflamed periodontal tissue.

Macrophage colony-stimulating factor signaling is important in inflammation driven bone resorption. Osteotropic factors stimulate M-CSF expression in a verity of cell types and promote osteoclast recruitment and bone resorption. In arthritic inflammation, locally produced TNF-α and IL-1β can further stimulate interleukin-7 expression from stromal cells, which in turn activate T-cells to generate M-CSF, whose signaling via c-Fms is significantly associated with active bone loss [28]. Interestingly, in this paper we show for the first time increased M-CSF expression in gingival fibroblasts in response to the pro-inflammatory cytokines TNF-α and IL-1β. The fact that gingival fibroblast produce both M-CSF and IL-34 in response to pro-inflammatory cytokines, and that these molecules can contribute to recruitment of bone resorbing osteoclasts further points at a pivotal role for gingival fibroblast in inflammation driven bone loss in periodontitis. Our time-course data indicate that IL-1β and TNF-α stimulated M-CSF expression peak already after 3–6 hours while IL-34 expression still increased up to 48 hours. This could indicate an acute role of M-CSF compared to IL-34 in inflammation-driven osteoclastogenesis.

The knowledge regarding the molecular pathways that govern expression of inflammatory mediators may have therapeutic significance in the management of inflammatory diseases including periodontitis and is therefore important to study. TNF-α and IL-1β activate two main signaling pathways, NF-κ*Β* and MAPK [17], [29]. In this paper we show that activation of these pathways are required for TNF-α and IL-1β to stimulate IL-34 which is in line with results in other cell types e.g. synovial fibroblasts [10], [11] and osteoblasts [30]. IL-34 could thus be a down-stream effector of the pro-inflammatory cytokines TNF-α and IL-1β and thereby induce osteoclast formation and bone resorption.

In conclusion, we show here for the first time that gingival fibroblasts express IL-34. The pro-inflammatory cytokines TNF-α and IL-1β regulate IL-34 expression in gingival fibroblasts, by a mechanism involving NF-κ*Β* and MAPK. This is the first study providing evidence for a possible role of IL-34 in periodontal disease, supported by its recently discovered role in other bone-degenerative diseases such as RA.
